# Unraveling the Multifaceted Role of Glutathione in Sepsis: A Comprehensive Review

**DOI:** 10.7759/cureus.56896

**Published:** 2024-03-25

**Authors:** Ratan Tandon, Ashish Tandon

**Affiliations:** 1 Medicine, Jawaharlal Nehru Medical College, Datta Meghe Institute of Higher Education and Research, Wardha, IND; 2 Pulmonology, Hari Daya Super Speciality Centre, Prayagraj, IND

**Keywords:** therapeutic intervention, organ dysfunction, immune modulation, oxidative stress, glutathione, sepsis

## Abstract

Sepsis remains a formidable challenge in healthcare, characterized by a dysregulated host response to infection, leading to organ dysfunction and high mortality rates. Glutathione, a critical antioxidant and regulator of cellular redox balance, has emerged as a key player in the pathophysiology of sepsis. This comprehensive review explores the multifaceted role of glutathione in sepsis, focusing on its involvement in oxidative stress, immune modulation, and organ dysfunction. Glutathione depletion exacerbates oxidative damage and inflammatory responses, thereby contributing to the progression of sepsis. Understanding the intricate mechanisms underlying glutathione dysregulation in sepsis offers potential therapeutic avenues, with strategies targeting glutathione pathways showing promise in mitigating septic complications. However, further research is needed to optimize therapeutic approaches and identify biomarkers for patient stratification. Overall, this review underscores the importance of elucidating glutathione's role in sepsis management to improve clinical outcomes and reduce the global burden of this life-threatening condition.

## Introduction and background

Sepsis is a life-threatening condition that arises when the body's response to infection leads to widespread inflammation, organ dysfunction, and, potentially, septic shock. It remains a significant healthcare challenge worldwide, with high mortality rates despite advances in medical care [1]. Glutathione, a tripeptide antioxidant in all mammalian cells, is critical in maintaining cellular redox balance, detoxification, and immune function. In sepsis, disruptions to glutathione homeostasis have been implicated in the pathogenesis of oxidative stress, immune dysregulation, and organ dysfunction. Understanding the intricate role of glutathione in sepsis could uncover novel therapeutic targets and strategies for improving patient outcomes [2].

This review aims to comprehensively examine the multifaceted role of glutathione in sepsis. By synthesizing existing literature, we seek to elucidate how glutathione influences critical pathways in sepsis pathophysiology, including oxidative stress, immune response, and organ dysfunction. Additionally, we aim to evaluate the therapeutic potential of targeting glutathione in sepsis management and identify gaps in current knowledge for future research directions.

## Review

Glutathione: an overview

Structure and Function of Glutathione

Glutathione features a distinctive gamma peptide linkage connecting the carboxyl group of the glutamate side chain and cysteine, with the cysteine residue bound to glycine. This unique molecular structure confers stability to glutathione, shielding it from peptidase degradation [3]. Glutathione biosynthesis entails two adenosine triphosphate (ATP)-dependent stages: initially, γ-glutamyl cysteine is formed from L-glutamate and L-cysteine, facilitated by glutamate-cysteine ligase. Subsequently, glycine is incorporated to generate glutathione, a reaction catalyzed by glutathione synthetase. Although all animal cells can produce glutathione, its synthesis in the liver is pivotal for overall glutathione production [4]. Glutathione is the most abundant non-protein thiol in animal cells, distributed within the cytosol and organelles. Over 90% of cellular glutathione is found in its reduced form (GSH), with the remaining portion in the oxidized form (GSSG). However, the systemic availability of orally ingested glutathione is constrained due to poor bioavailability [5].

Glutathione assumes a crucial role as an antioxidant, countering reactive oxygen species and safeguarding cells against oxidative harm. It engages in thiol protection, regulates the redox state of cellular proteins, and manages cellular thiol metabolism amid oxidative stress conditions. Additionally, glutathione aids detoxification by conjugating with electrophiles and diminishing oxidants [6]. Beyond its antioxidant functions, glutathione participates in redox signaling and facilitates post-translational thiol modifications of proteins during oxidative stress. The interconversion between the reduced (GSH) and oxidized (GSSG) states is orchestrated by nicotinamide-adenine dinucleotide phosphate (NADPH) and catalyzed by enzymes such as glutathione reductase [7].

Cellular Distribution and Regulation

Nuclear distribution: Research indicates that glutathione undergoes translocation into the nucleus during the initial stages of the cell cycle. This relocation of glutathione to the nucleus has been associated with various cellular processes and facilitates progression through the cell cycle [8]. Understanding glutathione dynamics within the nucleus provides insights into its roles in DNA replication, repair, and gene expression regulation, thereby highlighting its significance in fundamental cellular functions.

Subcellular localization: While predominantly found in the cytosol, glutathione also exhibits compartmentalization within cellular organelles such as mitochondria, peroxisomes, and nuclei. The redox status and sources of glutathione within these compartments vary, with nuclear glutathione predominantly existing in its reduced form and serving pivotal roles in preserving proteins crucial for DNA repair and transcriptional regulation [9]. The diverse subcellular localization of glutathione underscores its versatility in modulating distinct biochemical pathways and cellular functions across different organelles.

Regulation and transport: Glutathione biosynthesis primarily occurs in the cytosol, yet emerging evidence suggests an active involvement of glutathione transport in regulating subcellular redox equilibrium. The transport of glutathione across intracellular membranes is facilitated by specific transporters responsible for its import and export, thereby contributing to the fine-tuning of subcellular glutathione levels [10]. Understanding the intricate mechanisms governing glutathione transport and its regulatory roles provides valuable insights into cellular redox homeostasis and its implications for health and disease.

**Figure 1:**
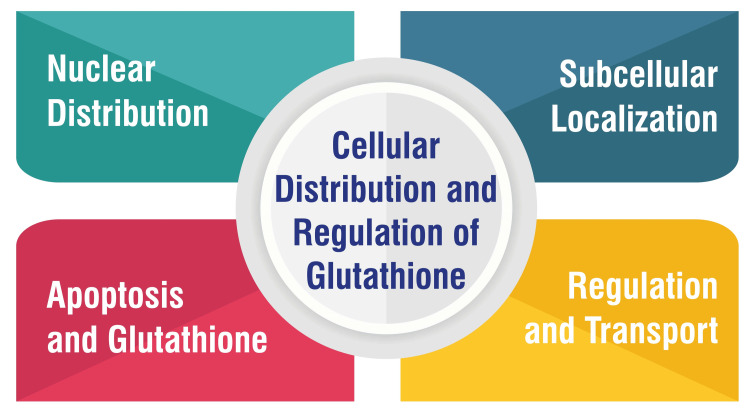
Cellular distribution and regulation Image Credit: Ratan Tandon

Apoptosis and glutathione: Depletion of glutathione is a hallmark of apoptotic cell death induced by diverse stimuli. Alterations in intracellular glutathione levels, particularly the ratio of reduced glutathione (GSH) to oxidized glutathione (GSSG), exert regulatory effects on apoptosis by modulating protein modifications through glutathionylation and influencing redox signaling pathways [11]. Elucidating the interplay between glutathione dynamics and apoptotic signaling pathways offers potential therapeutic avenues for manipulating cell fate decisions and managing diseases characterized by dysregulated cell death processes. Cellular distribution and regulation are shown in Figure 1.

Antioxidant and Redox Properties

Antioxidant role: Glutathione is renowned for its potent antioxidant properties and is synthesized ubiquitously throughout the body in high concentrations within cells. It is a crucial component of the antioxidant defence system, detoxifying electrophilic xenobiotics and modulating redox-regulated signal transduction pathways [12]. The multifaceted antioxidant functions of glutathione play pivotal roles in maintaining cellular homeostasis and protecting against oxidative damage, contributing to overall health and well-being.

Oxidative stress prevention: Alongside other antioxidants such as N-acetyl-cysteine, vitamins A, E, and C, glutathione is pivotal in mitigating oxidative stress by scavenging free radicals within cells [12]. By counteracting the harmful effects of oxidative stress, glutathione contributes to the reduction of various disease risks, including cancer, Parkinson's disease, Alzheimer's disease, and numerous others. Its involvement in oxidative stress prevention underscores its significance in disease prevention and management.

Redox buffer: As a physiologic redox buffer, glutathione is critical in maintaining cellular redox balance by donating thiol electrons to neutralize harmful oxidants and reversing oxidative damage to biomolecules [13]. Through reversible oxidation of critical protein cysteine residues via S-glutathionylation, glutathione regulates redox signaling pathways, modulating cellular responses to oxidative stress and maintaining cellular homeostasis.

Redox status visualization: Innovative studies have utilized targeted redox sensors to visualize the redox status of cytosolic glutathione, offering insights into the unique redox environment near organelle membranes and transmembrane redox gradients [13]. These advancements provide valuable information about the dynamic balance between oxidants and antioxidants within cells, facilitating a deeper understanding of cellular redox regulation and its implications for health and disease.

Glutathione redox status: Research has unveiled alterations in glutathione redox homeostasis among adolescents affected by obesity and anemia [14].

**Figure 2:**
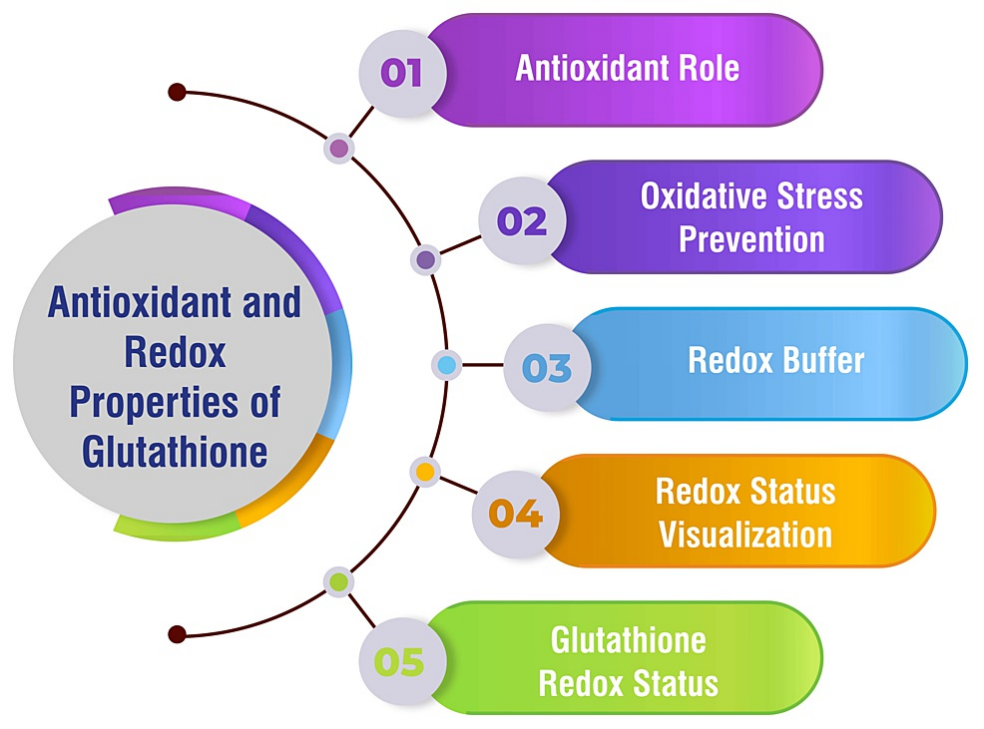
Antioxidant and redox properties image Credit: Ratan Tandon

Elevated GSH: GSSG ratios and increased glutathione peroxidase activity observed in subjects with obesity and anemia suggest a compensatory increase in redox defence mechanisms to counteract oxidative burden. These findings highlight the body's adaptive responses to oxidative stress and underscore the importance of glutathione in maintaining redox balance and cellular integrity. The antioxidant and redox properties of glutathione are shown in Figure 2.

Glutathione and oxidative stress in sepsis

Glutathione Depletion in Sepsis

Depletion of glutathione in sepsis can have profound implications. Studies indicate that glutathione is pivotal in cellular defenses against oxidative and nitrosative stress during sepsis, characterized by a pronounced redox imbalance [15,16]. In the acute phase of sepsis, there is an upsurge in the synthetic capacity of glutathione in the liver and other tissues, underscoring its critical significance in this condition [16]. However, hepatic glutathione levels experience a notable reduction within six hours of sepsis onset, indicating a heightened susceptibility to oxidative stress among septic patients [15]. Factors influencing glutathione synthesis in sepsis encompass cysteine availability and the activity of the enzyme glutamate cysteine ligase, both pivotal for maintaining optimal glutathione levels [15,16]. Glutathione depletion may impede neutrophil infiltration and foster bacterial proliferation. In contrast, supplementation with glutathione precursors like N-acetyl-L-cysteine can bolster neutrophil infiltration, curb bacterial proliferation, and enhance survival rates [16]. This underscores the criticality of maintaining adequate glutathione levels to bolster an effective response to sepsis.

Consequences of Glutathione Depletion

Lower levels of glutathione heighten susceptibility to oxidative stress, diminishing the body's capacity to counteract oxidative damage effectively. Consequently, this can lead to cellular impairment and dysfunction [17]. Glutathione's pivotal role in fundamental cellular processes, including cell differentiation, proliferation, and apoptosis, underscores its significance in maintaining cellular homeostasis. Imbalances in glutathione levels can disrupt these processes, contributing to the onset and progression of various diseases [17]. Moreover, glutathione deficiency has been implicated in a spectrum of human ailments spanning cancer, age-related diseases, cystic fibrosis, cardiovascular disorders, inflammatory conditions, immune dysregulation, metabolic disorders, and neurodegenerative diseases [17].

**Figure 3:**
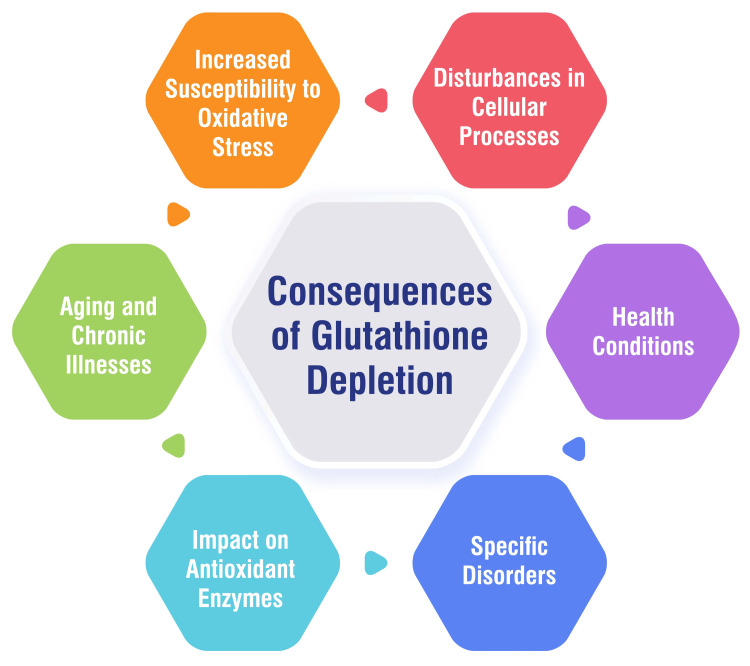
Consequences of glutathione depletion Image Credit: Ratan Tandon

Specific disorders stemming from disturbances in glutathione metabolism, such as glutathione synthetase deficiency, can precipitate conditions like compensated hemolytic anemia and other associated health complications [17]. The depletion of glutathione may adversely impact the activity of crucial antioxidant enzymes like glutathione peroxidase and glutathione reductase, potentially compromising the body's ability to neutralize harmful free radicals and maintain redox equilibrium [18]. Furthermore, decreased activity of enzymes involved in glutathione metabolism has been correlated with age-related chronic illnesses, accentuating the importance of sustaining optimal glutathione levels for overall health and well-being [18]. The consequences of glutathione depletion are shown in Figure 3.

Relationship Between Glutathione and Oxidative Stress Markers

The relationship between glutathione and oxidative stress markers is intricate and context-dependent. In plants, glutathione often serves as a marker of oxidative stress, reflecting its crucial role in antioxidant defense systems [19]. Conversely, glutathione is an important oxidative stress marker in cancer, essential for preserving antioxidant defenses and detoxifying harmful substances within cells [20]. Maintaining the balance between the reduced (GSH) and oxidized (GSSG) forms of glutathione is paramount for effective redox control, signal transduction, and gene regulation, all of which are perturbed in various diseases [21]. Studies have demonstrated that exposure to stress can induce changes in glutathione levels and redox state, leading to fluctuations in the GSH/GSSG ratio and total glutathione concentrations over time [19]. The dynamics of glutathione responses to stress may involve initial decreases followed by subsequent increases or further oxidation as stress escalates [19]. Emphasizing the importance of assessing glutathione levels alongside other components of the antioxidative defense system underscores its relevance in understanding stress avoidance strategies and alternative protection pathways [19]. This highlights the intricate interplay between glutathione and oxidative stress markers, underscoring the need for comprehensive analysis to elucidate their roles in various physiological and pathological contexts.

Glutathione and immune response in sepsis

Modulation of Immune Cell Function by Glutathione

Glutathione is pivotal in modulating immune cell function, particularly in sepsis and infectious diseases, contributing significantly to the body's defense mechanisms. Studies have indicated that glutathione supplementation can bolster cellular redox status, thereby bolstering the immune response against infections [22]. Crucially, glutathione is indispensable for regulating the delicate balance between innate immunity and inflammation, a balance critical for effectively combating infections and averting organ failure, as seen in conditions like sepsis [23].

Glutathione's support of immune system function extends to its influence on T-cell lymphocytes, pivotal players in the immune response, and its control over free radicals essential for inflammatory processes [23]. In the immune response against tuberculosis, glutathione emerges as a critical regulator, impacting the activation, metabolism, cytokine release, redox activity, and free radical levels of macrophages, natural killer cells, and T cells [24]. Maintaining optimal glutathione levels proves crucial for enhancing immune responses against tuberculosis infection, thereby improving outcomes for patients with heightened susceptibility, such as those with HIV or type 2 diabetes [24].

In essence, glutathione serves as a vital immunomodulatory antioxidant by stabilizing redox activity, steering cytokine profiles toward a beneficial immune response, and augmenting the function of T lymphocytes [24]. This underscores the profound significance of glutathione in modulating immune cell function and bolstering immune responses in diverse infectious and inflammatory conditions, highlighting its potential as a therapeutic target for enhancing host defenses.

Influence of Glutathione on Cytokine Production

In sepsis, glutathione has emerged as a significant determinant of cytokine production. Studies indicate that glutathione exhibits potent anti-inflammatory effects by reducing the levels of pro-inflammatory cytokines such as TNF-α and IL-1β [25]. Moreover, glutathione-induced immune-stimulatory activity fosters the polarization of M1-like macrophages, potentially facilitated by ROS-scavenging antioxidants, thus influencing cytokine production and immune responses [26]. Furthermore, glutathione is critical in finetuning the balance between innate immunity and inflammation during sepsis. It bolsters the immune system by supporting T-cell lymphocytes and regulating free radicals essential for the inflammatory healing process [23]. However, glutathione deficiency can compromise immune cell performance, limiting their ability to mount protective immune responses and heightening susceptibility to oxidative stress-induced damage [23].

Impact of Glutathione on Immune Cell Apoptosis

Glutathione depletion is a pivotal factor in immune cell apoptosis, particularly notable in lymphoid cells, where it serves as an early hallmark observed during the apoptosis process [27]. Intriguingly, studies reveal intricate regulation of glutathione levels and sensitivity to apoptosis, with apoptosis defects potentially contributing to a spectrum of diseases, including cancer, autoimmune disorders, and acquired immune deficiency syndrome [28]. This glutathione depletion is a standard feature of apoptotic cell death initiated by various stimuli such as stress, environmental agents, and cytotoxic drugs [11]. Such depletion can instigate alterations in the intracellular milieu conducive to apoptosis promotion, thereby contributing to the progression of cell death [11]. Moreover, glutathione plays a significant role in orchestrating cancer cell demise by modulating sensitivity to cytotoxic drugs, ionizing radiation, and cytokines while also influencing DNA synthesis, cell proliferation, and death [29]. It is worth noting that glutathione depletion aligns with apoptosis and extends to other forms of cell death, underscoring its crucial role in regulating cellular mechanisms dictating cell fate [29]. This elucidates the multifaceted involvement of glutathione in cellular processes, emphasizing its pivotal role in orchestrating cell survival and demise.

Glutathione and organ dysfunction in sepsis

Role of Glutathione in Protecting Against Organ Injury

Glutathione assumes a vital role in safeguarding against organ injury by actively preventing oxidative stress. It functions as an antioxidant by intercepting ROS and engaging with various free radicals, including hydroxyl radicals, thereby thwarting cellular damage [30]. The paramount importance of glutathione in human disease is widely acknowledged, emphasizing its pivotal role in upholding cellular health and functionality [30]. In sepsis and multiple organ failure, glutathione serves multifaceted functions, encompassing the scavenging of toxic ROS, detoxifying exogenous toxic compounds like drugs, and regulating of protein metabolism [31]. Reduced glutathione concentrations have been documented in skeletal muscle samples from intensive care unit (ICU) patients, showcasing a correlation between glutamine depletion and mortality rates, thereby underscoring the potential implications of glutathione depletion in critical illness scenarios [31]. The cytoprotective effects of glutathione are extensive and indispensable for cellular defense mechanisms against oxidative stress and other detrimental factors [31]. The role of glutathione in shielding against organ injury holds paramount significance due to its antioxidant properties and capacity to regulate cellular redox balance. This positions glutathione as a crucial contributor in mitigating oxidative damage and preserving cellular health amidst conditions such as sepsis and critical illness [15].

Glutathione and Endothelial Dysfunction

Glutathione plays a pivotal role in combatting endothelial dysfunction in sepsis by shielding against oxidative damage and microangiopathic dysfunction induced by heightened endothelial-generated hydrogen peroxide (H2O2) levels [32]. Failure to buffer cellular H2O2 can precipitate oxidative stress and microangiopathic dysfunction, thereby contributing to the pathophysiology of septic shock [32]. Notably, genetic disparities in glutathione levels and age-related declines have been documented, potentially compromising H2O2 neutralization and predisposing individuals to adverse outcomes in sepsis [32]. Emerging research suggests that glutathione can reverse endothelial dysfunction and enhance nitric oxide bioavailability, crucial for sustaining vascular health in sepsis [33]. The antioxidant properties of glutathione assume critical significance in scavenging ROS and detoxifying harmful compounds, thereby underscoring its cytoprotective effects across various tissues, including the endothelium [31]. In the milieu of sepsis, where redox imbalance looms large, the preservation of optimal glutathione levels emerges as pivotal for upholding endothelial function and averting organ dysfunction associated with this condition [15].

Implications for Specific Organs

Sepsis presents as a condition characterized by a significant surge in the number of obstructed capillaries, culminating in organ hypoxia, which can elicit detrimental effects across various bodily organs [34]. Organ dysfunction stands as a hallmark of sepsis, with the liver serving as a quintessential example of sepsis-associated excretory dysfunction. Notably, approximately 20% of jaundice admissions are attributed to sepsis, underscoring its profound impact on liver function [35]. The emergence of organ dysfunction is a crucial distinguishing factor between sepsis and uncomplicated infections, accentuating the pivotal role of organ impairment in delineating sepsis [35].

In sepsis, impairment within the cardiovascular system, gastrointestinal tract, and central nervous system is pervasive, exerting a significant influence on patient outcomes. While therapeutic interventions targeting specific organs, such as beta-blockers for the cardiovascular system, early enteral nutrition for the gastrointestinal tract, and light sedation/early rehabilitation for the central nervous system, have exhibited promising potential, their efficacy remains limited [35]. Future research endeavors aim to explore novel organ-specific strategies grounded in a comprehensive understanding of pathophysiology to enhance outcomes for septic patients [35].

Organ dysfunction in sepsis transcends mere consequences of diminished tissue oxygen delivery; it involves intricate mechanisms encompassing endothelial and microvascular dysfunction, immune dysregulation, and cellular metabolic reprogramming. Targeting these mechanisms holds promise for affording organ protection and augmenting survival rates among septic patients [36]. Moreover, discerning these mechanisms' adaptive or maladaptive nature and pinpointing phase-specific biomarkers emerge as critical strides toward refining therapeutic approaches and bolstering patient outcomes in sepsis management [36].

Therapeutic potential of glutathione in sepsis

Strategies to Replenish Glutathione Levels

Dietary choices: Incorporating sulfur-rich foods into one's diet, such as broccoli, cauliflower, garlic, onions, eggs, nuts, legumes, fish, and chicken, can significantly boost the body's natural production of glutathione [37]. Additionally, certain produce like avocado, asparagus, spinach, squash, melons, grapefruit, and peaches contain a plant-based form of glutathione that the body can convert to replenish its supply [38]. Furthermore, vegetables like broccoli, cauliflower, and cabbage contain compounds that stimulate the body's glutathione levels and support essential liver detoxification processes [38].

Supplementation: Supplementation offers another avenue to support glutathione levels. N-acetylcysteine (NAC), a precursor to cysteine, an amino acid vital for glutathione synthesis, has shown promise in preserving and replenishing glutathione levels [39]. Moreover, whey protein, containing cysteine, has been linked to promoting glutathione synthesis and reducing oxidative stress [39].

Lifestyle changes: Adopting certain lifestyle habits can also influence glutathione levels. Limiting alcohol consumption can help protect the body's ability to produce glutathione and reduce undue strain on its resources [38]. Ensuring adequate sleep is crucial for maintaining optimal glutathione levels and overall health [39]. Additionally, regular physical activity not only supports mental and physical well-being but also has the potential to reduce oxidative stress and sustain glutathione levels [40].

Glutathione Precursors and Analogs as Potential Therapies

Glutathione precursors and analogs have emerged as promising therapeutic candidates across various conditions, including sepsis, to bolster cellular glutathione levels and replicate its protective effects [41,42]. Strategies involving these compounds encompass the utilization of GSH monoethyl ester, GSH esters, cysteinyl-modified GSH derivatives, cysteine prodrugs, and GSH codrugs [41,43]. Extensive research has explored their potential in elevating cellular glutathione levels and combating oxidative stress in various ailments, including neurodegenerative disorders, cystic fibrosis, viral infections, aging, cancer progression, and chemoresistance [43,44]. Ongoing efforts are dedicated to pinpointing effective GSH analogs or precursors capable of engendering molecules with akin cellular protective effects as glutathione. This avenue of research holds immense promise for unveiling novel therapeutic avenues across diverse health conditions through the modulation of glutathione levels and its associated enzymes [42]. Furthermore, the quest for GSH precursors and analogs that enhance pharmacological properties and bioavailability is imperative for their therapeutic efficacy, particularly in conditions like multiple sclerosis [45].

Clinical Trials and Evidence Supporting the Use of Glutathione

Clinical trials and evidence underscore the therapeutic promise of glutathione in sepsis, elucidating its pivotal role in combatting oxidative stress and inflammation, hallmark features of this condition [15,46]. Glutathione, a tripeptide endowed with antioxidant properties, plays a vital role in scavenging reactive oxidant species, detoxifying harmful compounds, and regulating protein metabolism [31]. Studies reveal an upsurge in glutathione turnover during the acute phase of sepsis, accentuating its active engagement in cellular defenses against infection [47]. Factors influencing glutathione synthesis in sepsis encompass cysteine availability and the activity of the enzyme glutamate cysteine ligase, both integral for glutathione production [15]. Research indicates a heightened synthesis of glutathione across various tissues during the acute phase of sepsis, underscoring its significance in counteracting the redox imbalance characteristic of this condition [15,46]. Impediments in glutathione synthesis may arise due to cysteine depletion, protein-energy malnutrition, hyperglycemia, and pharmacologic doses of glucocorticoids [47,15]. Antioxidant therapies employing substances like vitamin C, E, N-acetylcysteine, and melatonin have been proposed as adjunctive treatments for a septic shock to address oxidative stress and inflammation [32]. Overall, the collective evidence substantiates the therapeutic potential of glutathione in sepsis, emphasizing its pivotal role in fortifying cellular defense mechanisms against the deleterious effects of oxidative stress and inflammation.

## Conclusions

This review delved into glutathione's intricate role in sepsis, highlighting its multifaceted contributions to the disease process. Our exploration shows that glutathione plays a pivotal role in maintaining cellular redox balance, modulating immune responses, and protecting against organ dysfunction during septic insults. Depletion of glutathione levels exacerbates oxidative stress, fuels inflammatory cascades, and compromises cellular function, thereby exacerbating the severity of sepsis. The implications of these findings are profound, offering insights into potential therapeutic strategies for managing sepsis. Targeting glutathione pathways presents a promising avenue for intervention, with the potential to mitigate oxidative damage, attenuate inflammatory responses, and improve clinical outcomes in septic patients. However, further research is needed to elucidate the optimal approaches for glutathione supplementation or modulation and to identify biomarkers for patient stratification. Ultimately, understanding the complex interplay between glutathione and sepsis pathophysiology is key to developing more effective treatment modalities and reducing the burden of this life-threatening condition on global health.
